# The tumor immune microenvironment of thyroid cancer and colorectal cancer: cellular crosstalk and therapeutic implications

**DOI:** 10.3389/fimmu.2026.1916192

**Published:** 2026-09-04

**Authors:** Guangzhe Zhang, Chengzhong Xing, Yuehua Gong

**Affiliations:** 1 Department of Thyroid Surgery, First Hospital of China Medical University, Shenyang, Liaoning, China; 2 Department of Anus and Intestine Surgery, First Affiliated Hospital of China Medical University, Shenyang, Liaoning, China; 3 Tumor Etiology and Screening Department of Cancer Institute and General Surgery, The First Hospital of China Medical University, Shenyang, China

**Keywords:** cellular crosstalk, colorectal cancer, immunotherapy, single-cell sequencing, thyroid cancer, tumor immune microenvironment

## Abstract

Thyroid cancer (TC) and colorectal cancer are common malignancies of the endocrine and gastrointestinal systems, respectively, and continue to impose a substantial global disease burden. The tumor immune microenvironment (TIME) plays a central role in tumor initiation, progression, and therapeutic response; however, the cellular composition and intercellular communication networks within the TIME exhibit marked heterogeneity between these two malignancies. Recent advances in single-cell sequencing and spatial transcriptomics have revealed complex and dynamic alterations in immune cell subsets, stromal cell functions, cytokine networks, and metabolic programs within the TIME. In particular, bidirectional communication between tumor cells and immune cells has emerged as a critical determinant of immune evasion and therapeutic resistance. Nevertheless, most existing studies have focused on individual tumor types, and systematic comparisons of the shared and distinct features of the TIME in thyroid cancer and colorectal cancer remain limited. Moreover, combination therapeutic strategies targeting the TIME are still under active investigation. This review systematically compares the immune microenvironments of thyroid cancer (TC) and colorectal cancer (CRC), highlighting the development of more precise and effective immunotherapeutic strategies for thyroid cancer and colorectal cancer.

## 1 Introduction

The tumor immune microenvironment, comprising tumor cells, immune cells, stromal cells, and extracellular matrix components, critically regulates tumor progression, immune escape, and therapeutic response. In thyroid cancer, the TIME is generally characterized by an immunosuppressive phenotype with enrichment of tumor-associated macrophages (TAMs) and regulatory T cells (Tregs), particularly in aggressive subtypes such as anaplastic thyroid carcinoma (ATC) (1). Distinct histological subtypes, including papillary, follicular, and anaplastic thyroid cancers, exhibit marked heterogeneity in immune-cell infiltration, immune checkpoint expression, and metabolic features (2, 3). ATC frequently shows abundant TAM infiltration and elevated PD-L1 expression, whereas the low prevalence of microsatellite instability further limits the applicability of immune checkpoint blockade in most thyroid cancers (4). By contrast, CRC exhibits a strong association between immune architecture and microsatellite status. MSI-H tumors generally display high mutational burden and abundant tumor-infiltrating lymphocytes and respond favorably to PD-1/PD-L1 blockade, whereas microsatellite-stable (MSS) CRC is commonly characterized by T-cell exclusion and enrichment of Tregs, myeloid-derived suppressor cells, and TAMs (4).

Despite these differences, thyroid cancer and CRC share several mechanisms of immune suppression. TAMs and Tregs produce immunosuppressive mediators such as IL-10 and TGF-β and cooperate with inhibitory immune checkpoint pathways to restrain antitumor immunity (5–7). Cellular crosstalk is further shaped by cytokine and chemokine networks, metabolic competition, and oncogenic signaling pathways. Thyroid cancer frequently exhibits enhanced glycolysis and glutamine metabolism, whereas CRC displays broader metabolic plasticity involving glycolysis, lipid metabolism, and amino-acid utilization (8). In addition, PI3K/AKT/mTOR and Wnt/β-catenin signaling can regulate immune-cell recruitment, checkpoint expression, and immune exclusion (9, 10). Recent advances in single-cell sequencing and spatial transcriptomics have enabled high-resolution characterization of these intercellular networks (11, 12). This review summarizes the cellular composition and crosstalk mechanisms of the TIME in thyroid cancer and CRC and discusses their implications for biomarker-guided immunotherapy and rational combination strategies.

## 2 Tumor immune microenvironment in thyroid cancer and colorectal cancer

### 2.1 Diversity and functional roles of immune cell subsets

The tumor immune microenvironment of thyroid cancer exhibits prominent immunosuppressive features, characterized particularly by the enrichment of TAMs and Tregs (13). TAM infiltration is relatively abundant in thyroid cancer, especially in aggressive histological subtypes such as ATC and medullary thyroid carcinoma (MTC) (1). In parallel, the infiltration of CD8^+^ T cells is often limited, and a substantial proportion of these cells display an exhausted phenotype, thereby restricting their capacity to mount effective antitumor immune responses (14). The antitumor functions of B cells and natural killer (NK) cells may also be impaired within the thyroid cancer microenvironment, further weakening immune surveillance (15). In CRC, by contrast, the density and spatial distribution of CD8^+^ T cells within tumor tissues are strongly associated with patient survival and represent important prognostic indicators (16, 17). The CRC microenvironment also contains abundant myeloid-derived suppressor cells (MDSCs) and Th17 cells, and disruption of the Th17/Treg balance has been implicated in tumor progression and unfavorable clinical outcomes (18). Importantly, the application of single-cell RNA sequencing (scRNA-seq) has revealed previously underappreciated immune-cell states and subpopulations in both thyroid cancer and CRC (19, 20). These include functionally distinct B-cell subsets expressing immunoregulatory or immune checkpoint-associated molecules, highlighting a level of immune heterogeneity that cannot be adequately captured by conventional lineage-based classification systems (21). These findings indicate that differences in the abundance, activation state, and spatial distribution of immune-cell subsets contribute substantially to the distinct immune landscapes of thyroid cancer and CRC.

### 2.2 Roles of stromal cells in the tumor microenvironment

Stromal cells, particularly cancer-associated fibroblasts (CAFs), are critical regulators of the TIME in both thyroid cancer and CRC (22). In thyroid cancer, CAFs actively participate in extracellular matrix (ECM) remodeling through the secretion of TGF-β, MMPs, and other soluble mediators (23). Such remodeling not only promotes tumor invasion and metastatic dissemination but may also generate a physical and biochemical barrier that restricts immune-cell infiltration into tumor tissues. CAFs exhibit even greater functional heterogeneity in CRC, where distinct subpopulations, including inflammatory CAFs and myofibroblastic CAFs, can exert markedly different effects on tumor biology (24, 25). Inflammatory CAFs may secrete chemokines such as CCL2, thereby recruiting monocytes and TAMs and reinforcing an immunosuppressive microenvironment (26–28). Other CAF subsets contribute to ECM deposition, TGF-β signaling, immune exclusion, and treatment resistance (29, 30). Thus, CAFs function as a collection of functionally specialized cell states that dynamically interact with immune and malignant cells. Endothelial cells also play an important role in shaping the TIME of both malignancies (24, 31). Tumor-associated endothelial cells participate in aberrant angiogenesis, resulting in structurally and functionally abnormal vasculature (32). These abnormal vessels can impair the trafficking and extravasation of effector T cells into the tumor parenchyma while simultaneously contributing to tissue hypoxia and acidosis. Hypoxic and poorly perfused regions further enhance immunosuppressive signaling and metabolic stress, thereby creating conditions that favor tumor progression and immune escape (33, 34). Consequently, stromal cells and tumor vasculature constitute active components of the immunoregulatory network rather than passive structural elements of the tumor microenvironment.

### 2.3 Regulation by the extracellular matrix and physical properties of the microenvironment

**Figure 1:**
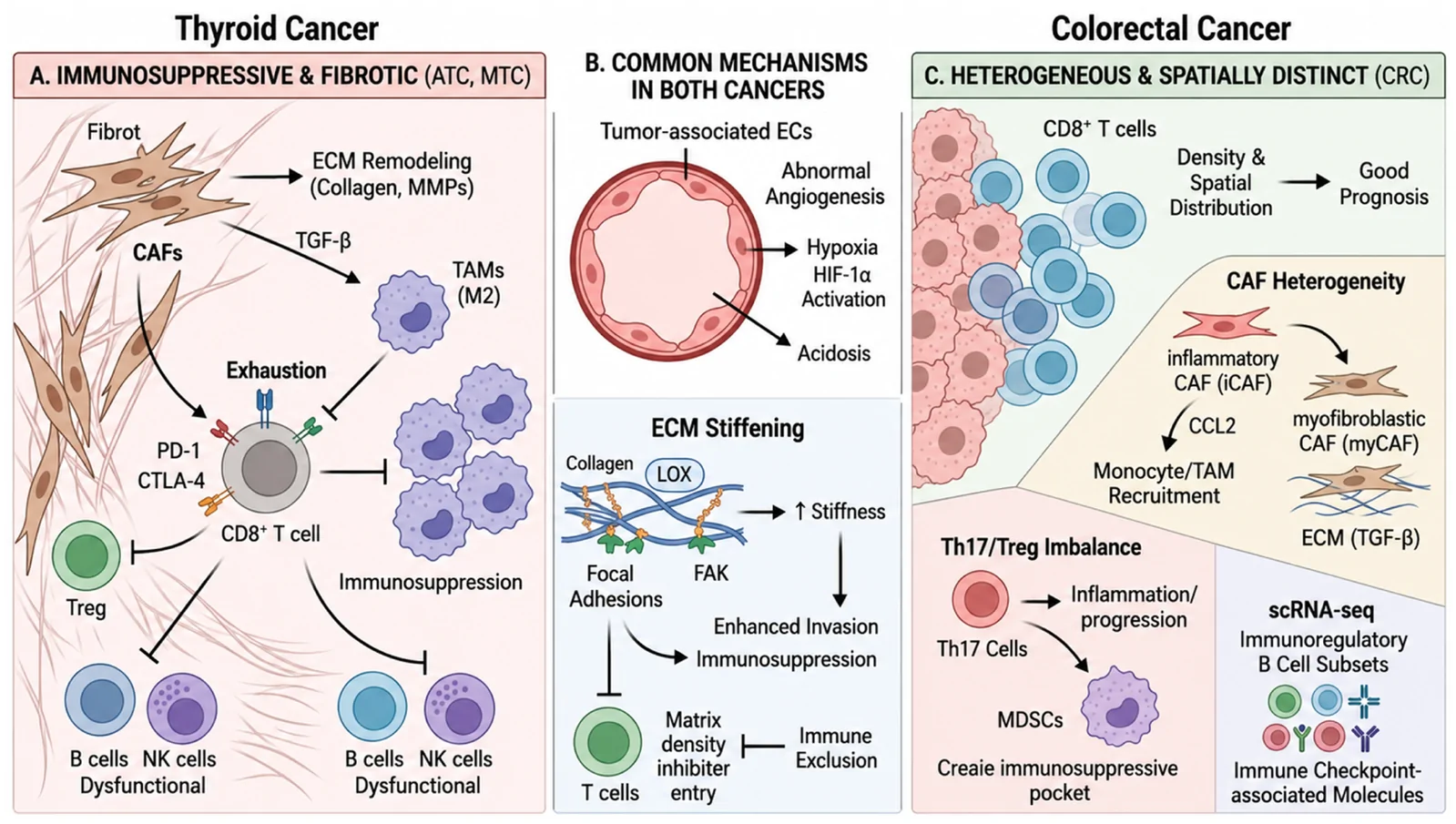
Tumor immune microenvironment in thyroid cancer and colorectal cancer. **(A)** Immunosuppressive microenvironment in thyroid cancer. **(B)** Common mechanisms in both cancers. **(C)** Immunosuppressive microenvironment in colorectal cancer.

The physical and biochemical properties of the extracellular matrix are increasingly recognized as major determinants of tumor progression and immune regulation in thyroid cancer and CRC (35). Excessive deposition of ECM components, including collagen and hyaluronan, can increase matrix density and interstitial pressure within tumors (36). Elevated interstitial pressure may compress intratumoral blood vessels, reduce perfusion, and aggravate tissue hypoxia. In turn, hypoxia activates HIF-1α-dependent transcriptional programs that promote tumor-cell survival, invasion, angiogenesis, and the production of immunosuppressive mediators (37, 38). Increased matrix stiffness also has direct biological consequences. Mechanotransduction through integrins and focal adhesion kinase (FAK) can enhance tumor-cell migration and invasive potential while simultaneously influencing the phenotype of surrounding stromal and immune cells (39, 40). In particular, a stiffened ECM may favor the acquisition of immunosuppressive macrophage phenotypes and contribute to the establishment of a feed-forward loop in which stromal remodeling and immune suppression reinforce one another (35, 39). Such changes may also restrict the physical penetration of cytotoxic lymphocytes into tumor nests, thereby contributing to immune-excluded phenotypes. ECM-remodeling enzymes represent another important component of this regulatory axis (22, 41). Lysyl oxidase (LOX), which promotes collagen crosslinking and matrix stiffening, has been implicated in the generation of fibrotic and immune-excluded tumor microenvironments (42). Increased activity of LOX and related matrix-modifying enzymes may therefore promote tumor progression through both mechanical and immunological mechanisms (43). These observations indicate that differences in stromal architecture, matrix composition, and mechanical properties may contribute substantially to the distinct patterns of immune infiltration and therapeutic responsiveness observed in thyroid cancer and CRC (**Figure 1**).

## 3 Immunosuppressive mechanisms of cellular crosstalk

### 3.1 Crosstalk between the immune checkpoints

Within the tumor microenvironment of thyroid cancer and CRC, the interaction between programmed cell death protein 1 (PD-1) and its ligand PD-L1 represents one of the central mechanisms underlying tumor immune escape (44, 45). Thyroid cancer cells, particularly those in papillary thyroid carcinoma (PTC) and ATC, frequently exhibit elevated PD-L1 expression (46). Engagement of PD-L1 with PD-1 on tumor-infiltrating T cells suppresses T-cell receptor (TCR) signaling, reduces the production of effector cytokines such as IFN-γ and TNF-α, and promotes T-cell dysfunction or apoptosis, thereby weakening antitumor immunity (19, 47, 48). In CRC, in addition to the PD-1/PD-L1 axis, other co-inhibitory receptors, including lymphocyte activation gene 3 (LAG-3) and T-cell immunoglobulin and mucin-domain containing-3 (TIM-3), are frequently co-expressed on exhausted T cells (49–51). Their simultaneous activation establishes a multilayered inhibitory receptor network that further reinforces T-cell dysfunction (49, 52). Crosstalk between tumor cells and dendritic cells also contributes to immune suppression. Tumor-derived inhibitory signals can impair DC maturation and antigen-presenting capacity, thereby limiting effective priming of tumor-specific T cells (53, 54). In parallel, activation of indoleamine 2,3-dioxygenase (IDO)-mediated tryptophan metabolism depletes local tryptophan and promotes the accumulation of kynurenine, creating a metabolically unfavorable environment for effector T cells while favoring immune tolerance (55, 56). Therefore, immune checkpoint signaling and metabolic suppression converge to impair antigen presentation, induce T-cell anergy, and establish a robust immunosuppressive barrier within the TME.

### 3.2 Cytokine- and chemokine-mediated immunosuppressive circuits

Cytokines and chemokines act as major intercellular messengers that coordinate immunosuppressive networks in both thyroid cancer and CRC (57, 58). In thyroid cancer, TAMs and Tregs contribute to the immunosuppressive cytokine milieu, partly through the production of IL-10 and TGF-β (59). These cytokines suppress dendritic cell maturation, inhibit NK cell cytotoxicity, and promote the differentiation and maintenance of immunosuppressive Tregs, thereby generating a self-reinforcing suppressive circuit (60, 61). TGF-β additionally contributes to immune exclusion by modulating stromal activation and extracellular matrix remodeling, linking immune suppression with changes in tissue architecture (62). In CRC, IL-6-mediated signaling is an important component of the immunosuppressive network. IL-6 activates STAT3, thereby promoting the expansion and functional activation of MDSCs (63). These cells suppress T-cell responses through mechanisms including arginase-1 expression, reactive oxygen species (ROS) production, and depletion of metabolites required for T-cell activation and proliferation (64–66). Chemokine networks further determine the composition of the immune infiltrate. In thyroid cancer, the CCL17/CCL22-CCR4 axis contributes to the recruitment of Tregs, whereas in CRC, CXCR2-dependent signaling promotes the accumulation of neutrophils and granulocytic MDSCs (67–70). These tumor-specific chemokine programs contribute to distinct immunosuppressive landscapes while converging on the common endpoint of impaired antitumor immunity.

### 3.3 Interplay between tumor metabolism and immune cell function

**Figure 2:**
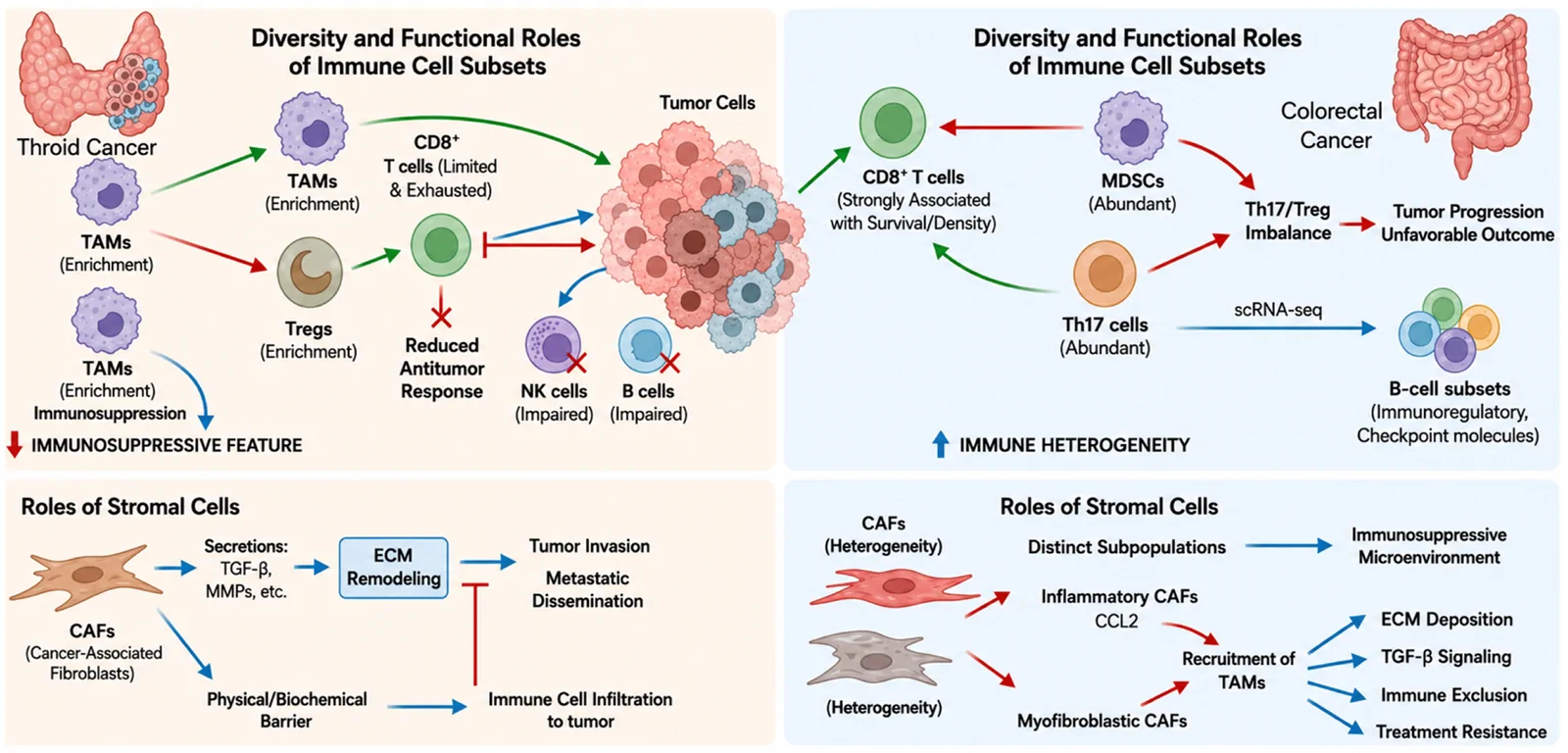
Immunosuppressive mechanisms of cellular crosstalk in thyroid cancer and colorectal cancer.

Metabolic reprogramming of tumor cells is closely intertwined with immune dysfunction and represents a major mechanism through which the TME becomes immunosuppressive (71, 72). In thyroid cancer, increased expression of glucose transporter 1 (GLUT1) and enhanced aerobic glycolysis enable tumor cells to consume large amounts of glucose. This creates intense metabolic competition within the TME and limits glucose availability for effector T cells, which depend on glycolytic metabolism to sustain proliferation, cytokine production, and cytotoxic activity (61). Consequently, glucose deprivation can impair T-cell activation and promote functional exhaustion. In CRC, upregulation of lactate dehydrogenase A (LDHA) promotes lactate production and accumulation (73, 74). Elevated extracellular lactate suppresses T-cell and NK-cell function and can promote macrophage polarization toward an immunosuppressive M2-like phenotype, thereby reinforcing local immune tolerance (75–77). Lactate-mediated signaling may therefore act at multiple levels, directly weakening cytotoxic lymphocytes while simultaneously reprogramming myeloid cells toward tumor-promoting states (78). Tryptophan metabolism represents another shared immunoregulatory pathway in both malignancies. IDO and tryptophan 2,3-dioxygenase (TDO) catalyze tryptophan degradation and increase the production of kynurenine (79, 80). Kynurenine can activate the aryl hydrocarbon receptor (AhR), which favors Treg differentiation and suppresses effector T-cell responses (81, 82). Thus, metabolic competition and tumor-derived metabolites are not merely consequences of rapid tumor growth but active regulators of immune-cell fate and function. Collectively, these metabolic–immune interactions form an integrated network that supports immune escape and may provide therapeutically actionable vulnerabilities in thyroid cancer and CRC (**Figure 2**).

## 4 Combination immunotherapeutic strategies targeting cellular crosstalk

### 4.1 Comparative efficacy of immune checkpoint inhibitors in thyroid cancer and colorectal cancer

The clinical efficacy of ICIs differs markedly between CRC and thyroid cancer, largely reflecting differences in the composition and functional state of their TIMEs (83). In CRC, microsatellite instability-high (MSI-H) status is one of the most established biomarkers for predicting response to ICIs (84). Anti-PD-1 monotherapy achieves objective response rates of approximately 30–40% in patients with MSI-H CRC, whereas response rates in MSS disease remain below 5% (85). This discrepancy is largely attributable to the limited immune-cell infiltration and prominent T-cell exclusion observed in MSS CRC, which may be driven in part by activation of pathways such as Wnt/β-catenin signaling (86, 87). In thyroid cancer, pembrolizumab has demonstrated durable disease control in a subset of patients with advanced disease, particularly in tumors with high PD-L1 expression or elevated tumor mutational burden (88). Although the overall tumor mutational burden of thyroid cancer is generally low, ATC may exhibit relatively higher genomic instability, which could partly explain the clinical activity observed in selected patients (89, 90). Dual immune checkpoint blockade, such as combined PD-1 and CTLA-4 inhibition, has the potential to further enhance T-cell activation in both tumor types; however, this approach is accompanied by an increased risk of immune-related adverse events (91). Therefore, biomarker-guided patient stratification based on microsatellite status, PD-L1 expression, tumor mutational burden, and other TIME-associated features remains essential for optimizing the clinical use of ICIs.

### 4.2 Targeting tumor-associated macrophages and myeloid-derived suppressor cells

TAMs and MDSCs are major immunosuppressive populations within the TIME and represent attractive targets for combination immunotherapy (92–94). In thyroid cancer, inhibition of CSF-1R can reduce TAM abundance and may shift the remaining macrophage population toward a more pro-inflammatory, antitumor phenotype, thereby facilitating recovery of CD8^+^ T-cell function (95, 96). Strategies aimed at reprogramming macrophages rather than simply depleting them are also being investigated, including approaches based on trained immunity and metabolic modulation (26). In CRC, targeting the CCL2/CCR2 axis can reduce the recruitment of monocytic MDSCs and immunosuppressive monocytes into tumors, while CXCR2 blockade may inhibit the trafficking of granulocytic MDSCs and neutrophil-like suppressor populations (97, 98). These interventions can enhance T-cell-mediated antitumor immunity and may improve responsiveness to PD-1 blockade, particularly in CRC (99, 100). In addition, metabolic pathways that sustain MDSC suppressive activity, such as fatty acid oxidation and PI3K-dependent signaling, have emerged as potential therapeutic targets (101, 102). By disrupting the recruitment, survival, or suppressive function of myeloid populations, these strategies may help remodel an immune-excluded TIME into a more immunologically permissive state.

### 4.3 Adoptive cell therapy and tumor vaccines for remodeling the tumor microenvironment

Adoptive cell therapies and therapeutic cancer vaccines can directly enhance antitumor immune responses and may contribute to TIME remodeling; however, their efficacy is often constrained by local immunosuppressive mechanisms. In CRC, CAR-T therapy faces several barriers, including TGF-β-rich stromal niches, hypoxia, and the dense extracellular matrix produced by CAFs (103–105). Engineering CAR-T cells to resist TGF-β signaling, for example through expression of dominant-negative TGF-β receptors, may improve their persistence and effector function within the hostile tumor microenvironment (106). In thyroid cancer, tumor-infiltrating lymphocyte (TIL)-based approaches have shown potential in selected patients with advanced disease. Ex vivo expansion of tumor-reactive lymphocytes can restore effector function, although their persistence after reinfusion may require additional cytokine support (107). Other lymphocyte populations, including γδ T cells, NK cells, and NKT-like cells, may also participate in antitumor responses and provide additional opportunities for cell-based therapies (108–110). Therapeutic vaccines represent another strategy for enhancing tumor-specific immunity. Dendritic-cell vaccines loaded with tumor-associated antigens, such as thyroglobulin in thyroid cancer or carcinoembryonic antigen in CRC, can induce antigen-specific CD8^+^ T-cell responses (111–113). More recently, personalized neoantigen vaccines generated through whole-exome sequencing and HLA-binding prediction have enabled the design of patient-specific multiepitope vaccines (107, 114). Nevertheless, local immune suppression within the TIME remains a major obstacle to durable vaccine efficacy. Combining tumor vaccines or adoptive cell therapies with ICIs or stromal-targeting agents may therefore be required to achieve sustained antitumor responses.

### 4.4 Combined metabolic modulation and tumor microenvironment remodeling

Metabolic reprogramming is a major driver of immune suppression within the TIME, making metabolic pathways attractive targets for combination therapy. Metformin activates AMPK and can suppress tumor-cell glycolysis and lactate production, thereby potentially improving the metabolic fitness of antitumor immune cells (115, 116). In both thyroid cancer and CRC, modulation of tumor glucose metabolism may reduce the competitive metabolic pressure imposed on T cells and attenuate the suppressive activity of myeloid cells (117, 118). Tryptophan metabolism is another important therapeutic target. Inhibition of IDO aims to restore local tryptophan availability while reducing kynurenine accumulation, thereby alleviating T-cell metabolic dysfunction and immune tolerance (55, 72, 79, 80). Although clinical outcomes of IDO-targeted approaches have been variable, the underlying pathway remains mechanistically relevant and may require improved biomarker selection or more rational combination strategies. Hypoxia also contributes to immune dysfunction and abnormal angiogenesis. Targeting HIF-1α signaling or angiogenic pathways may normalize tumor vasculature, reduce hypoxia, and facilitate the infiltration of effector lymphocytes (119). In thyroid cancer, tyrosine kinase inhibitors targeting VEGFR and related angiogenic pathways may therefore exert immunomodulatory effects in addition to their direct antitumor activity (120, 121). Similarly, in CRC, metabolic interventions targeting lipid accumulation or fatty acid utilization may reduce M2-like TAM polarization and suppressive myeloid-cell function (75). These findings support the concept that metabolic therapy should be considered not only as a tumor-cell-directed intervention but also as a strategy for reshaping the TIME (**Supplementary Table 1**).

## 5 Conclusion

The tumor immune microenvironment (TIME) is a dynamic and highly heterogeneous ecosystem that critically shapes tumor progression, immune escape, and therapeutic response in both thyroid cancer and CRC. Despite their distinct histological and molecular characteristics, these malignancies share several immunosuppressive features, including enrichment of tumor-associated macrophages and regulatory T cells, impaired effector T-cell activity, abnormal stromal remodeling, and metabolic competition within the tumor niche. Cellular crosstalk mediated by immune checkpoints, cytokines, chemokines, and metabolic pathways further reinforces immune dysfunction and contributes to resistance to immunotherapy. Current evidence indicates that therapeutic efficacy depends not only on targeting tumor cells but also on disrupting the immunosuppressive networks established by myeloid cells, stromal cells, abnormal vasculature, and tumor metabolism. Accordingly, rational combinations integrating immune checkpoint blockade with TAM/MDSC targeting, metabolic modulation, anti-angiogenic therapy, adoptive cell therapy, or therapeutic vaccination may offer greater clinical benefit than single-agent strategies. Importantly, substantial interpatient and intratumoral heterogeneity necessitates biomarker-guided treatment selection. Advances in single-cell RNA sequencing and spatial transcriptomics provide powerful tools for resolving immune and stromal cell states, defining spatially organized cellular niches, and reconstructing cell–cell communication networks. Future studies integrating single-cell, spatial, genomic, metabolic, and clinical data will be essential for identifying dominant immunosuppressive circuits and developing mechanism-based combination therapies. Ultimately, a deeper understanding of TIME heterogeneity and cellular crosstalk may enable the transition from empirical immunotherapy toward precise, individualized treatment strategies for thyroid cancer and CRC.
